# Cooperative Virtual Reality Gaming for Anxiety and Pain Reduction in Pediatric Patients and Their Caregivers During Painful Medical Procedures: Protocol for a Randomized Controlled Trial

**DOI:** 10.2196/63098

**Published:** 2025-03-31

**Authors:** Stefan Liszio, Franziska Bäuerlein, Jens Hildebrand, Carolin van Nahl, Maic Masuch, Oliver Basu

**Affiliations:** 1 Center for Virtual and Extended Reality in Medicine University Hospital Essen Essen Germany; 2 Pediatric Health Play Team Center for Child and Youth Medicine University Hospital Essen Essen Germany; 3 Technical University of Denmark Copenhagen Denmark; 4 Entertainment Computing Group University of Duisburg-Essen Duisburg Germany

**Keywords:** virtual reality, extended reality, mixed reality, serious game, video game, pain, anxiety, stress, child, caregiver, patient experience, well-being, medical procedures, punctures, distraction, intervention

## Abstract

**Background:**

The hospital experience is often marked by fear and pain, particularly for children undergoing medical procedures. Sedation is commonly used to alleviate patient anxiety, but it poses additional health risks. Caregivers, usually the parents, also experience emotional distress during the child’s hospital stay, which can further exacerbate the child’s anxiety and pain. While various interventions exist to ease patient distress, few consider the emotional well-being of caregivers.

**Objective:**

This study aims to explore the effectiveness of a cooperative virtual reality (VR) game as a novel nonpharmacological solution to reduce anxiety and pain for both pediatric patients and their caregivers during medical procedures. Specifically, we aim to investigate whether the VR game “Sweet Dive VR” (SDVR), designed for children aged between 6 and 12 years to play with 1 caregiver, can alleviate anxiety and pain during different types of needle punctures and Kirschner-wire removal.

**Methods:**

A prospective multicenter randomized clinical trial will be conducted. Eligible participants will be identified by scanning the hospital information system, and group allocation will follow stratified randomization. During the medical procedure, patients in the VR condition will play SDVR with a caregiver present, while patients in the control group will listen to a recording of gently crashing waves. Data collection will be carried out through self-reports of patients and caregivers using visual analog scales and questionnaires at 2 measurement time points: before and after the intervention. In addition, observation by the interviewers will occur during the intervention to capture emotional and pain reactions as well as interaction quality between patients and caregivers and smoothness of the procedure flow using a structured observation protocol. The measured variables will encompass patient affect and pain, caregiver affect, player experience, patient experience, and the flow of the procedure.

**Results:**

As of November 2024, we enrolled 39 patients and caregivers, 28 of whom completed the study. Data collection is still ongoing.

**Conclusions:**

Cooperative VR gaming, as exemplified by SDVR, emerges as a promising intervention to address anxiety and pain in pediatric patients while involving caregivers to support the emotional well-being of both parties. Our approach strives to foster positive shared experiences and to maintain trust between children and caregivers during emotionally challenging medical situations.

**Trial Registration:**

German Clinical Trial Register (DRKS) DRKS00033544; https://drks.de/search/en/trial/DRKS00033544

**International Registered Report Identifier (IRRID):**

DERR1-10.2196/63098

## Introduction

### Background

Fear and pain characterize the hospital experience. Children, due to a lack of understanding of the situation, often show extreme anxiety reactions even in objectively harmless situations such as magnetic resonance imaging [1]. Hence, the patient’s anxiety and stress can complicate treatment procedures and interfere with the healing process [2-4]. As a result, it is a common practice to sedate patients by administering medication. However, the use of such medication is associated with additional health risks [5], particularly in the context of child development [6]. An additional factor that can influence the emotional experience of child patients is their caregivers, in most cases a parent. Caregivers are also under extraordinary emotional pressure during the child’s stay in hospital. If a medical procedure causes intense fear or pain, it can strain the caregiver-child relationship, especially if the child perceives the caregivers as siding with medical staff instead of supporting them—for example, by helping to physically restrain the child [7]. As a result, anxiety, stress, and pain of patient and caregiver can mutually depend on and reinforce each other [8]. While there are numerous pharmacological and nonpharmacological options to ease the patient, the experience of the caregivers is rarely the focus of such interventions.

Therefore, it is advisable to explore nonpharmacological solutions to reduce anxiety and pain. Distracting patients from the medical situation in a controlled manner is an approach that has proven successful for a large variety of media such as movies, music, or toys [9]. The use of immersive technologies such as digital games and virtual reality (VR), in particular, has been shown to be effective in reducing anxiety and pain in a variety of medical situations in numerous studies, especially for needle-related procedures, burn care, and in dental medicine [9-13]. However, these solutions typically address only the patient, which may lead to patients and caregivers feeling disconnected from each other. VR has the potential to result in patients feeling separated from their caregivers due to the sensory shielding provided by wearing a head-mounted display (HMD), which is necessary to create the desired level of immersion. This, in turn, could have an unintended anxiety-increasing effect.

### Approach

We are pursuing a new approach in our work: we have developed the cooperative, asymmetrical VR game “Sweet Dive VR” (SDVR) that involves the patient and a caregiver equally in the gameplay and promotes joint communication [14]. In this way, both parties are distracted from the intimidating medical situation without being artificially separated from each other. Involving caregivers in the game, thereby creating positive shared player experiences, can be a way of maintaining children’s trust and reducing feelings of helplessness for both children and caregivers [15]. In addition to the game-related interaction with the child, the caregivers are given the task of reacting to certain steps of the medical procedure (eg, the application of a disinfectant or an oxygen cannula) by triggering in-game events. In this way, we achieve synchronization of the virtual world with the patient’s real-world sensory impressions, which is intended to preserve and strengthen immersion for the patient on the one hand, while supporting the caregivers’ sense of agency. SDVR was designed for children aged between 6 and 12 years and for application during needle-related procedures, in particular port punctures, bone marrow punctures, central and peripheral venous catheterization, and venipunctures. However, we are also planning to test our approach with more painful procedures, such as Kirschner-wire removal.

### Objectives

While several different works have successfully used VR games as a distraction during painful procedures [14-16], SDVR is, to the best of our knowledge, the first game that involves both the patient and the caregiver and aims to enhance the emotional experience of both. This study aims to evaluate the effectiveness of using cooperative VR games during medical procedures to improve patient and caregiver experiences. Specifically, we examine whether SDVR can reduce patients’ anxiety, pain, and agitation; improve cooperation; and positively influence caregivers’ emotional states compared to a control condition in which a calming atmosphere is created by playing natural ocean wave sounds to promote relaxation.

## Methods

### Hypotheses

In this study, we investigate whether targeted distraction from anxiety- and pain-inducing medical procedures through cooperative VR gaming can reduce experienced pain and anxiety while fostering well-being for both the patients and their caregivers. A list of our hypotheses is presented in Textbox 1. Hence, the primary outcome of the study is a reduction in pain and anxiety ratings of the patient (hypothesis 1) as well as an improvement in the caregiver’s emotional experience (hypothesis 2). Our intervention relies on targeted distraction through sensory immersion for the patient as well as cognitive immersion and game fun for the patient and caregiver. Thus, distraction from the medical procedure represents a further end point of the study (hypothesis 3 and hypothesis 5).

The smooth execution of the medical procedure, characterized by the cooperativeness of the patient and caregiver, the needlessness of sedation or fixation, and an uninterrupted flow, will be assessed as a secondary end point (hypothesis 4). Moreover, this study aims to evaluate the quality of SDVR with respect to the player experience of both patient and caregiver (hypothesis 5).

**A list of hypotheses.**

**Hypothesis 1: patient affect and pain**

- Patients playing Sweet Dive VR (SDVR) during the procedure will report reduced levels of *anxiety* compared with those in the control group.
- Patients playing SDVR during the procedure will report reduced levels of *pain* compared with those in the control group.
- Patients playing SDVR during the procedure will exhibit decreased *motoric activity* compared with those in the control group.
- *Observer’s* ratings of *patient cooperation* during the procedure will be improved in the SDVR group compared with those in the control group.
- *Caregivers’* ratings of patient *pain* will be lower in the SDVR condition than in the control condition.
- *Caregivers’* ratings of patient *anxiety* will be lower in the SDVR condition than in the control condition.
- *Observers’* ratings of patient *pain* will be lower in the SDVR condition than in the control condition.
- *Observers’* ratings of patient *anxiety* will be lower in the SDVR condition than in the control condition.

**Hypothesis 2: caregiver affect**

- Caregivers in the SDVR group will experience more *positive emotions* during the procedure compared with those in the control group.
- Caregivers in the SDVR group will experience fewer *negative emotions* during the procedure compared with those in the control group.
- *Observer’s* ratings of *caregiver cooperation* during the procedure will be improved in the SDVR group compared with those in the control group.

**Hypothesis 3: player experience**

- The more positive the player experience of the patients in the SDVR group is, the lower will be the *patients'* intensity of *anxiety* during the procedure.
- The more positive the player experience of the patients in the SDVR group is, the lower will be the *patients'* intensity of *pain* during the procedure.
- The more positive the playing experience of the *caregivers* in the SDVR group, the less negative will their *emotional experience* be.

**Hypothesis 4: patient experience**

- In the SDVR condition, more patients will have a feeling of *being in control over the situation* than those in the control condition.
- In the SDVR condition, more patients will report being *distracted* from the procedure than those in the control condition.
- The number of *patients* reporting that SDVR reduced their *agitation* will be greater than those reporting that listening to ocean waves reduced their agitation.
- *Patients* in the SDVR condition will be more *surprised by the pain* than those in the control condition.

**Hypothesis 5: flow of the procedure**

- The *observers’* rating of the *overall quality of the procedure* will be higher in the SDVR condition than in the control condition.

As interviewing children about their emotional and physical experience is a methodological challenge due to various biases, and because we strive for an objective assessment of the patient’s and caregiver’s experience, we follow a comprehensive, holistic approach in our methodology and the instruments used. In addition to the patients’ and caregivers’ self-reported emotional experiences, we also ask the caregivers to assess the patient’s anxiety and pain. In addition, the interviewers use a structured observation protocol to assess the patient’s experience, the gaming situation, and the course of the medical procedure.

### Potential Confounding and Influencing Factors

The meta-analysis carried out by Eijlers et al [11] indicates that VR-based distraction from anxiety and pain is more efficacious in younger children than in older children. Age and gender influence the perception of pain and anxiety during medical procedures. Younger children tend to report more pain during procedures such as venipuncture [16]. In self-reporting, girls tend to report more pain than boys [17]. However, older children and boys tend to rate pain and anxiety differently compared to younger children and girls [17]. Age and parental predictions of distress are substantial predictors of pain and anxiety on blood tests [18]. Therefore, age and gender will be considered as possible influencing factors in the analysis.

Our intervention addresses a range of medical procedures that have shown to be highly anxiety- and stress-inducing in pediatric patients but can generally be performed without anesthesia. It is possible that patient anxiety and pain ratings differ for different procedures and that these perceptions influence the effect of our intervention.

Fundamental anxiety as a patient characteristic (ie, trait anxiety) is also a likely influencing factor for anxiety and pain ratings [19]. Furthermore, there is evidence in the literature of a link between perceived anxiety and postoperative recall of pain [20], which could influence patients’ retrospective assessment of their pain. Therefore, we will consider the assessment of the caregivers and the observation of the interviewers in our study.

A well-documented phenomenon that negatively impacts the VR gaming experience is simulator sickness, which includes symptoms such as nausea, dizziness, headaches, and eye strain. There is evidence that children are less likely to react to VR exposure with these negative symptoms [21], but there have been few studies on the possible relationship between age and different forms of visually induced motion sickness. Nevertheless, we will record any indications of simulator sickness before and after the intervention as the described symptoms may relate to patients’ general health state. We have elaborated more on that topic in the Methods section, under the Simulator Sickness subsection.

Previous hospital experience, particularly about the previous interventions of the same or similar nature, as well as needle-related phobias and trauma, can influence the patient’s emotional response to the medical situation and will therefore be documented during screening. The actual diagnosis will also be recorded to assess the overall psychological situation of the patient and caregiver.

### Ethical Considerations

This study was approved by the ethics committee of the medical faculty of the University of Duisburg-Essen (22-10873-BO). The ethics committees of the other study centers, Universitätsklinikum Hamburg-Eppendorf, Germany, Ethik-Kommission der Ärztekammer Hamburg (approval 2023-200739-BO-bet) and Ethikkommission des Universitätsklinikum Schleswig-Holstein, Lübeck, Germany (approval 2023-708), also saw no ethical or legal objections to this study.

All patients and caregivers will be fully informed about the background and procedure of the study during the recruitment process before they consent to participate in the study. Both the legal guardians and the children must give their written consent to participate in the study. They will be informed about the protection of their personal data and their digital rights in accordance with the European General Data Protection Regulation.

During the onboarding process, the collected data will be pseudonymized. An individual, random participant ID will be created during enrollment and stored with the patient ID separate from the survey answers. Once data collection is complete, the pseudonymization table will be deleted so that the data are anonymous from this point on. The children will be informed of the possibility that they might undergo the procedure in the control group without HMD and sedation or anesthesia. Participants in the control group will be allowed to try the game after completing the medical procedure.

### Study Design

The study presented here is a prospective multicenter randomized nonblinded clinical trial. This study refers to protocol version 19 (March 1, 2023), which is registered in the German Clinical Trials Register (DRKS00033544). Any changes to the protocol will be submitted as an amendment to the responsible ethics committee of the University Hospital Essen for approval and filed in the DRKS. Data collection will take place at the University Hospital Essen, the University Hospital Schleswig-Holstein in Lübeck, and the University Hospital Hamburg-Eppendorf, all 3 located in Germany. Patients in the VR condition will play the game SDVR with 1 caregiver during the medical procedure. The beneficial effects of VR and digital games in distracting from anxiety and pain compared to the standard of care (ie, no intervention for distraction or sedation) have been demonstrated in numerous studies [9,11-13]. Therefore, our study particularly focuses on the effect of the shared gaming experience and addresses both the patient and the caregiver as recipients of the distraction and mood-enhancing intervention. Furthermore, to avoid the results being biased simply by the patient’s experience of getting an “extra treatment” and additional attention from the medical staff during participation in the study, we will compare the experimental condition with a control condition in which the patients and caregivers will also receive special treatment. Acoustic relaxation techniques are straightforward and unobtrusive to implement in medical procedures, making it possible to address both patients and caregivers simultaneously. In contrast to guided imagery, which requires active mental engagement—often challenging in stressful or painful situations—nature sounds offer a passive form of relaxation. This passivity makes them more accessible and appealing across a range of ages and cognitive abilities. Another option could be passive music therapy; however, natural sounds present a more universally neutral and calming experience, as they bypass personal musical preferences and the emotional resonance often associated with music. Therefore, we chose to play a recording of ocean waves to foster a calming atmosphere during the procedure.

### Procedure

**Figure 1:**
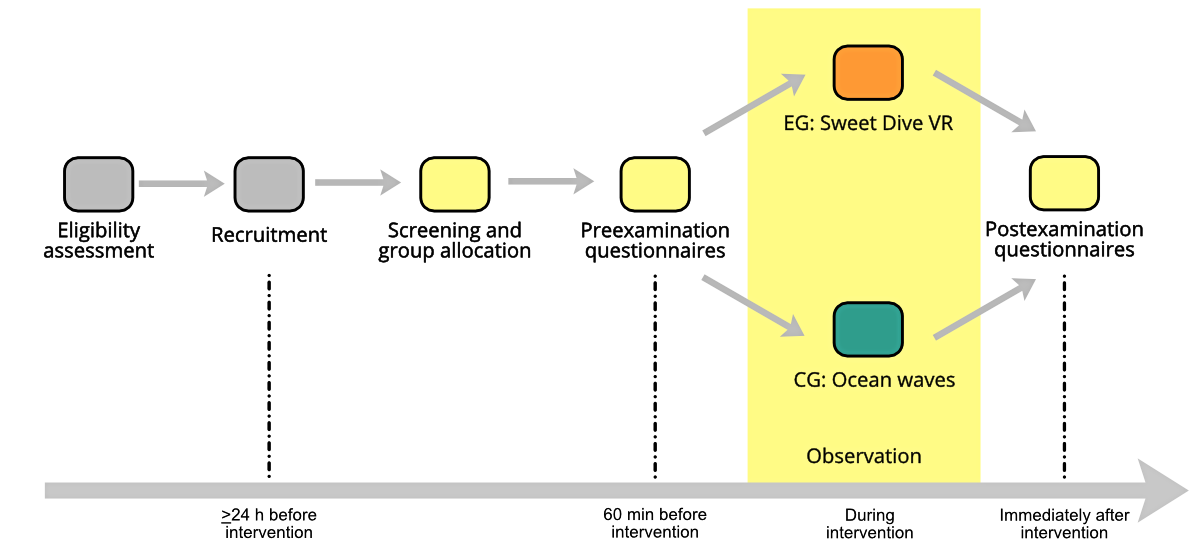
Study procedure timeline. Participants are assigned to either the experimental group (ie, Sweet Dive VR; SDVR) or the control group (ie, ocean waves). Pre- and postexamination questionnaires are administered to patients and caregivers. The interviewers assess the situation during the intervention using an observation protocol. CG: control group; EG: experimental group.

A diagram of the study procedure is shown in Figure 1. Patients meeting the eligibility criteria (Textbox 2) will be identified in the hospital information system. Patients and their caregivers will then be contacted by telephone or during their next stay in the hospital. They will be fully informed about the background and aim of the study, the procedure, and the 2 experimental conditions. Patients and caregivers will then have at least 24 hours to consider whether they want to participate. Refusals and their reasons will be recorded anonymously.

If they agree to participate, patients and caregivers will be asked to give their written consent. During registration, the patients will then complete the screening questionnaire together with an interviewer, based on which the randomized, stratified group allocation will be automatically performed. All questionnaires aimed at patients will be completed together with the interviewers. The task of the interviewers is to read out the questions and items if necessary and to help with comprehension problems. The interviewers are trained accordingly so that they do not interfere with the patient’s response behavior and do not suggest answers.

The participants will be requested to arrive 60 minutes before the actual appointment. During this time, the patients and caregivers will fill out the preexamination questionnaires in parallel. The participants will be reminded once again that participation is voluntary and that they can withdraw their participation at any time.

**Participant inclusion and exclusion criteria.**

**Inclusion criteria**

- Age 6 to 12 years (applies for patients only)
- Procedure possible without sedation or anesthesia (applies for patients only)
- Sufficient knowledge of German (applies for patients and caregivers)
- Ability to hold and operate a game controller (applies for patients and caregivers)

**Exclusion criteria**

- Visual impairments (eg, lack of stereo vision and color blindness; applies for patients only)
- Severe cognitive impairments (as assessed by the caregivers and physicians; applies for patients and caregivers)
- Diagnosed with epilepsy (applies for patients only)

**Termination criteria**

- Desire to end participation (applies for patients and caregivers)
- Clear signs of severe simulator sickness (applies for patients only)
- Extreme pain (applies for patients only)
- Extreme anxiety (applies for patients only)
- Other adverse events (applies for patients and caregivers)

In the SDVR condition, patients and caregivers will receive a verbal, written, and illustrated explanation from the interviewer about the process, goal, and controls of the game as well as a brief introduction to the background story either as a short video or in the form of a short picture story. The caregivers will be instructed that they can start the game using the buttons on the controller and trigger events in the game that correspond to the treatment steps.

After the patients and caregivers have entered the examination room, in the SDVR group, the interviewer will help the patient put on the HMD and hand them the controller. The caregivers will receive the game controller and can then start the game session. In the control condition, the audio file will be played from a tablet or notebook.

We made special efforts to make the setup process as straightforward and quick as possible to enable noninvasive and seamless integration into the medical procedure. The VR app starts automatically as soon as the HMD is switched on (ie, the so-called “kiosk mode”). The necessary realignment of the perspective in the virtual world to the patient’s position can be triggered simply by pressing a button on the game controller. The technical setup process, including a functional test of all components (eg, Bluetooth connection to the game controller), takes just 5 minutes on average and can be prepared before the actual treatment. Setting up and adjusting the HMD for the patient and handing over the game controller to the caregiver takes a further 1 to 2 minutes on average.

During the procedure, the interviewer will observe the medical situation, the patient’s reactions, and the interaction between the patients and the caregivers. The observations will be documented in an observation protocol.

At the end of the intervention, the postexamination questionnaires will be completed by the patients and caregivers. Interruptions and premature terminations of the intervention will be recorded, and the reasons will be noted. Children in the control condition will be allowed to try out the game after the end of the experiment.

If a participant wishes to terminate the intervention or if one of the termination criteria described in Textbox 2 is met, the intervention will be discontinued immediately, and the participant will return to the standard of care, and a note will be made in the observation protocol.

### Participants

#### Sample

Patients requiring port puncture, lumbar puncture, peripheral or central venous catheter insertion, or Kirschner-wire removal will be enrolled, each with 1 caregiver. Given the complexity of the planned study setting and its integration into the clinical processes, we performed a sensitivity analysis to ensure an appropriate test strength and sample size. We anticipate that the recruitment of 78 patients and caregivers each is feasible. On the basis of a power analysis with the G*Power software [22] and starting from a 2-sided *t* test with a significance level of α=.05 and a test power of 80%, we determined that a medium-size effect of approximately Cohen *d*=0.64 can be achieved with a corresponding sample size of 39 participants per group. In view of these statistical parameters and considering the objectives of the study, we decided that recruiting 26 participants in each of the 3 participating study centers would provide an appropriate balance between practical feasibility and statistical power.

#### Randomization and Stratification

Assignment to the experimental conditions will be automated by a study management software. The recruiters will have no opportunity to influence the assignment at any time. Allocation will use the method of stratified randomization using a minimization algorithm. Consequently, the patient-related variables (ie, strata) used for sample stratification, are as follows: (1) trait anxiety (State-Trait Anxiety Inventory for Children–trait anxiety), (2) age, (3) type of intervention, (4) previous experience with the procedure, and (5) sex. The minimization algorithm attempts to allocate a new patient to a group in such a way that the difference between the 2 groups is minimal for each stratum. If no clear allocation can be achieved with this procedure, a random allocation will be made.

### Material

#### Experimental Condition: SDVR

**Figure 2:**
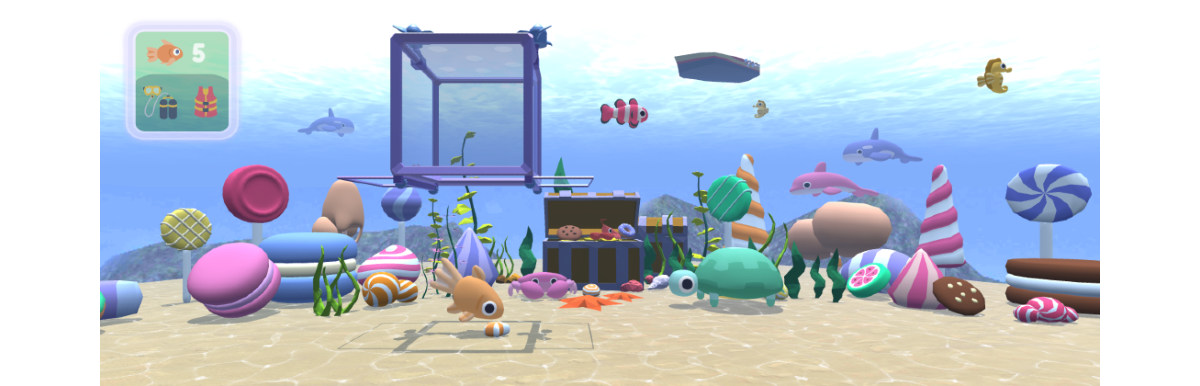
In “Sweet Dive VR,” the patient must attract a fish with candy and move the box down in the right moment to collect it. Next, the caregiver must move the box up again. The head-up display in the upper-left corner indicates the number of collected fish (top row) and events associated with the medical procedure triggered by the caregiver (bottom row).

The players’ mission will be to catch as many fish as possible for a medical examination after a cargo ship lost a container filled with candy. The patient and 1 caregiver will play the game cooperatively. The core mechanic of SDVR is attracting and catching fish. To attract a fish, the patient can drop a piece of candy into the water by pressing a button. A transport box moves horizontally on the upper edge of the screen from left to right and back. When the box is precisely above the fish, the patient must press another button at the right moment to bring it down and cover the fish. In the next step, the patient must inform the caregiver verbally so that the caregiver can press a button on the gamepad to move the box upward. The fish is then considered to be collected, and the process can start all over again. The number of fish collected will be shown to the VR player in a head-up display (Figure 2). The gameplay is infinite; that is, there is no fixed game end or goal. This way, we can respect the variable length of the treatment.

**Figure 3:**
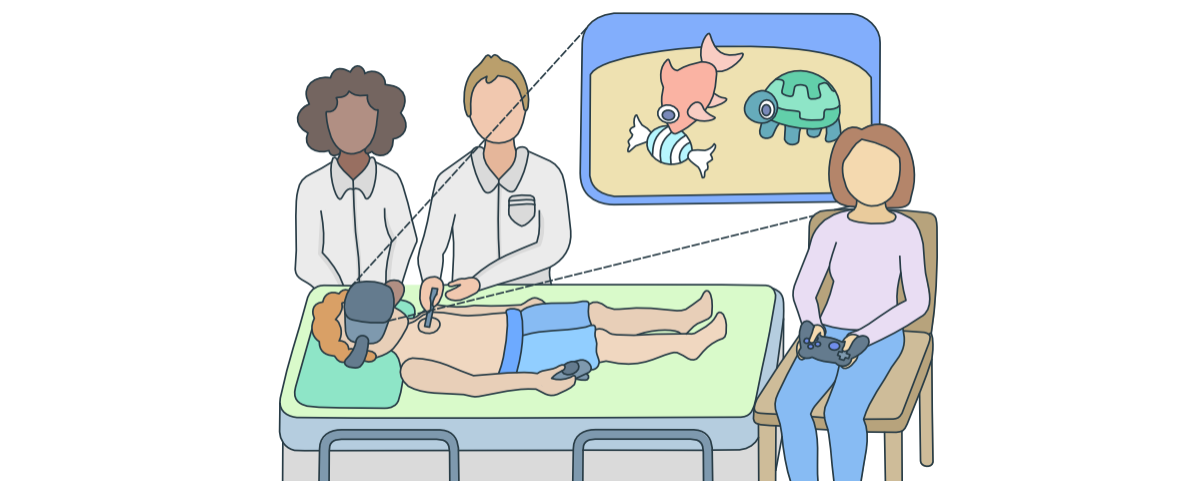
Schematic representation of the medical procedure and the patient-caregiver interaction in the asymmetric game design.

The game was designed to be played in a supine position (Figure 3). Consequently, the player’s perspective has been adjusted so that, while lying on their back, they not only look up at the virtual sky (ie, the virtual water surface as in the case of SDVR) but can also see the horizon and the ground. By slightly shifting the virtual camera, a natural visual impression is possible, suggesting that the player is standing. In numerous previous tests, we have not registered any negative effects such as simulator sickness.

The key feature of our game design is the inclusion of a caregiver in the gameplay as a second player. Through the asymmetrical game design [23], we can distract both the patient and caregiver from the treatment and create a shared positive experience. While both players must cooperate to pursue their shared objective (ie, catching the fish), they have different mechanics at their disposal to achieve that objective. However, not only are the actions available to the 2 players different but also the information about the game world is different. While the patient can see the game world via the HMD, the caregiver lacks all visual and auditory cues about the current game state (Figure 3). Thus, the caregiver is completely dependent on the communication of their child. Consequently, a high degree of concentration on the game events and the verbal communication of both players is required, which thus becomes an essential mechanic of the game and enhances the immersion of both players [14]. With the children giving verbal instructions to their caregivers, the game is designed to support their feeling of being in control over the situation. Moreover, the strong focus on communication between caregivers and children should enhance the sense of familiarity, security, and connectedness within the emotionally challenging situation. In addition to interacting with the child through the game, caregivers are assigned the task of responding to specific steps of the medical procedure (eg, the application of a disinfectant or an oxygen cannula) by triggering corresponding in-game events. This approach synchronizes the virtual world with the patient’s real-world sensory experiences, thereby preserving and enhancing the patient’s immersion. Concurrently, it grants caregivers a sense of agency and participation in the medical situation, reinforcing their involvement in the process.

As this study focuses on the description of the research protocol for the clinical evaluation of our approach, we refrain from delving deeper into the design rationale here and refer to the planned publication elsewhere.

#### Control Condition: Audio Recordings of Ocean Waves

The research field of environmental psychology explores the positive effects of natural environments on human experience [24], including in medical contexts [25,26]. It has been shown that exposure to natural environments can reduce acute and anticipatory stress. Annerstedt et al [27] showed a stress-recovery–promoting effect of birdsong in a virtual forest scenario. Liszio et al [28] have shown that the audiovisual presentation of virtual underwater environments significantly reduces acute and anticipatory stress. However, research on the effect of solely auditive presentation of nature sounds on the emotional experience of patients in medical situations is scarce; however, there is some evidence in the literature. Moreover, several studies indicate a significant reduction of patient anxiety and agitation during percutaneous coronary intervention [29] and coronary artery bypass graft surgery [30,31] when listening to nature sounds.

In the control condition, a 30-minute recording of ocean waves will be played instead of the VR game. Recording will start with a 1-minute introduction by the same soothing voice that narrates the companion character “Turtle” in SDVR. From then on, only the sound of the waves will continue.

#### Hardware

**Figure 4:**
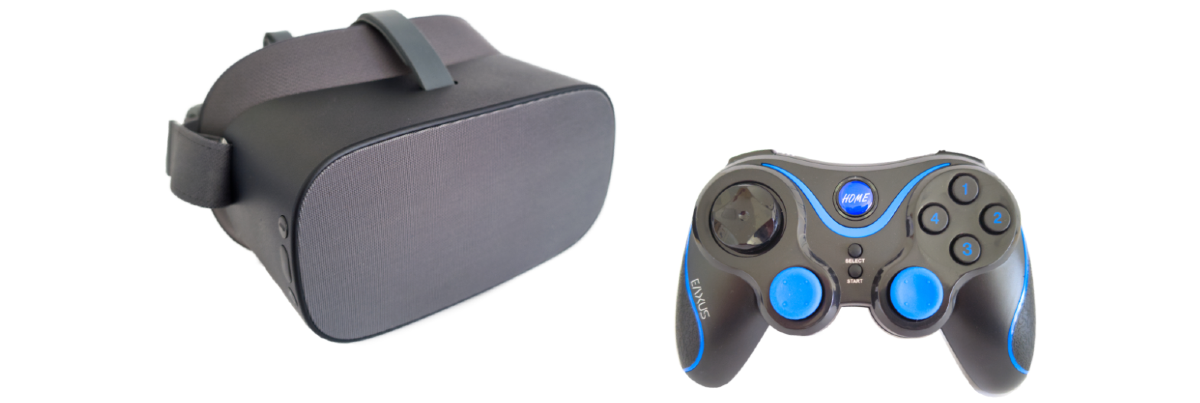
The Pico G2 4K head-mounted display with controller (left) and conventional gamepad (right) used in the experimental condition during the medical procedure.

Patients in the VR condition will play the game with a Pico G2 4K HMD (Figure 4, left). The device is a stand-alone HMD in which all calculations are performed on the device itself, so that no additional computer is required. The HMD has 3 df and can therefore only be used when sitting or lying down. User inputs are made via the associated controller, which resembles a simple remote control. A larger touchpad is located on the controller, which works like the directional pad on a typical game controller. In addition, the controller has a trigger button that is operated with the index finger. It can be controlled with 1 hand using either the left or right hand. We chose this hardware for the following reasons: the housing is comparatively easy to disinfect, as all external components are either hard plastic or rubber. Comparable devices from other manufacturers typically use foam covered with fabric for the face pad and elastic fabric for the headbands, which do not meet health care hygiene standards. Furthermore, no user account from third-party providers is required for operation. Apps can also be started in a so-called kiosk mode, that is, the corresponding app starts automatically when the device is switched on, which makes it much easier to use in the fast pace of everyday clinical practice. Finally, the standard controller’s small design and reduced number of buttons simplify operation with small hands. Children have no problems holding the controller and reaching all the buttons with their fingers. The symmetrical design allows operating the controller with either the left or right hand.

For the caregiver, we will use a conventional gamepad as an input device for the game. The gamepad and HMD are connected via Bluetooth. The advantage of using a common gamepad is that many people are familiar with the layout of the buttons and the handling while the acquisition costs are low.

For data collection, we will use tablet PCs. These will also be used to play the recording of ocean waves in the control condition.

### Psychometric Assessment

#### Emotional Experience

##### State and Trait Anxiety

The State-Trait Anxiety Inventory is an established questionnaire for measuring anxiety [32] (Table 1). It was developed to measure both state anxiety and the personality characteristics of anxiety (ie, trait anxiety). The questionnaire consists of 2 scales, one for assessing state anxiety and one for assessing trait anxiety. In this study, we will use the German translation provided by Laux et al [33].

For the child survey, we will use the German translation of the adapted State-Trait Anxiety Inventory for Children [19]. This adaptation is characterized by a reduced number of 20 per scale items and a simplified response scale as compared to the original State-Trait Anxiety Inventory. In addition, the rating options are reduced to 3, ranging from 1 (low anxiety) to 3 (high anxiety).

In addition, we will implement a visual analog scale (VAS), which provides an effective method of assessing children’s affective state as it offers a simple and easy to understand way of quantifying emotions. We will use the scale “Anxiety” by Gräßer et al [34]. This is based on a scale from 0 (not at all) to 10 (very strong). By simultaneously presenting an illustration the children can identify with and scaling the values from 0 to 10 on a line, children can express their emotional experience in a precise and differentiated way. For comparability between the children’s self-report and the impressions of the caregivers and the interviewers, we will also ask the caregivers and the interviewers to rate the patients’ pain perception on the same VAS.

**Table 1:** Breakdown of the psychometric instruments administered (ie, questionnaires, visual analog scales, and self-designed questions) for each respondent group and measurement time.

Respondents and measurement time			Psychometric instrument
**Children**			
	Screening	STAIC-T^a^	
	Preintervention measurement	STAIC-S^b^ VAS^c^ anxiety PANAS-K^d^ SSQ-C^e^	
	Postintervention measurement	STAIC-S VAS anxiety PANAS-K VAS pain SSQ-C Adapted PXI^f^ for childreng Patient experience Presence	
**Caregivers**			
	Preintervention measurement	PANAS PANAS-C-P^g^ VAS anxiety (child)	
	Postintervention measurement	PANAS 0-P VAS anxiety (child) VAS pain (child) PXI^h^ Player experienceg	
**Interviewers**			
	Observation	CHEOPS^i^ Self-designed questions for assessing procedure flow	

^a^STAIC-T: State-Trait Anxiety Inventory for children–trait anxiety.

^b^STAIC-S: State-Trait Anxiety Inventory for children–state anxiety.

^c^VAS: visual analog scale.

^d^PANAS-K: German translation of the Positive and Negative Affect Schedule for Children.

^e^SSQ-C: Child Simulator Sickness Questionnaire.

^f^PXI: Player Experience Inventory.

^g^PANAS-C-P: parent version of the Positive and Negative Affect Scale for Children.

^h^Sweet Dive VR only.

^i^CHEOPS: Children’s Hospital of Eastern Ontario Pain Scale.

##### Positive and Negative Emotions

To assess the participants’ affective state, we will use the positive affect negative affect schedule (PANAS) [35]. The questionnaire consists of 2 separate scales, one for positive affect (PA) and one for negative affect (NA). Each scale comprises a list of words that describe different emotions or moods. Respondents will be asked to indicate the extent to which they felt each of these words either “when thinking about the medical procedure” (before measurement) or “during the medical procedure” (after measurement).

For the caregivers, we will use the original questionnaire translated to German by Janke and Janke and Glöckner-Rist [36]. This version comprises 20 adjectives that describe emotional states. The rating scale describes the intensity with which these emotional states were experienced on a 5-point scale from 1 (not at all) to 5 (extremely). For the 2 dimensions PA and NA, the sum scores are formed from the intensity ratings of the corresponding items.

In addition to describing their own emotional experience, the caregivers assess the children’s emotional states using the parent version of the Positive and Negative Affect Scale for Children, which we will use in the German translation provided by Großheinrich [37]. The scale consists of 2 subscales to determine the children’s PA (12 items) and NA (15 items) according to the caregiver’s impression on a 5-point scale from 1 (very little or not at all) to 5 (extremely).

The participating children will be administered an adapted version of the original PANAS (PANAS-C) developed by Laurent et al [38]. To our knowledge, because no official German translation of the children’s version of the PANAS-C exists, we used this version as a starting point and compared the items with the German translations of the adjectives according to Janke and Glöckner-Rist [36]. As the PANAS-C comprises 30 items, rather than 20 items as in the version by Janke and Glöckner-Rist [36], additional adjectives for which no translation could be found were translated by the authors.

#### Pain

To measure the children’s perceived pain, we will use the VAS “Pain” by Gräßer et al [34], equal to the measurement of anxiety (as mentioned earlier). Again, we will ask the patients, the caregivers, and the interviewers for a rating.

The interviewers will rate the observed pain-associated behavior of the child using the Children’s Hospital of Eastern Ontario Pain Scale [39]. It can be used to monitor the effectiveness of interventions to reduce pain and discomfort in young children. The result is a pain score between 4 (no pain) and 13 (severe pain).

#### Player Experience

##### Overview

We will use the German translation of the Player Experience Inventory (PXI), which was developed by Graf et al [40], to assess the caregivers’ experiences while they play SDVR. The PXI consists of 10 dimensions that describe aspects of the player experience, allowing for a systematic analysis of the game design. Each dimension comprises 3 items, which are rated on a 7-point scale ranging from –3 (strongly disagree) to 3 (strongly agree).

For evaluating the caregivers’ experience, we will consider only the following constructs: “ease of control,” “goals and rules,” “challenge,” “meaning,” “curiosity,” and “mastery and immersion.” We will exclude the constructs “progress feedback,” “audiovisual appeal,” and “autonomy” from the evaluation of SDVR because the game does not have any audiovisual component for caregivers.

To date, there is no suitable and validated version of the PXI for children. As the PXI in its complete form is quite extensive and, in our experience, some item formulations are difficult for younger children to understand, we created a variant by selecting 1 item from each of the 10 dimensions that is clear and easy to understand. In the first round, 3 of the authors—an educator and health play specialist, a pediatric nurse with significant expertise in child-friendly medical language, and an entertainment computing specialist—independently selected 1 item per dimension that, in their opinion, met the criteria of representativeness for the dimension, child-appropriate concept complexity, and child-appropriate language. In the second step, the items for which there was no agreement between the 3 reviewers were discussed until consensus was reached. Moreover, we reduced the response options to a more child-friendly 5-point scale from −2 (strongly disagree) to +2 (strongly agree). It should be noted that this is a tentative, empirically unvalidated version. The data collected in the study may provide the basis for future efforts to develop a suitable player experience metric for children. For the sake of reproducibility and transparency, we included the final list of items in Multimedia Appendix 1.

The interviewers will be asked to assess the child’s player experience by rating the statements “The child had fun playing.”; “...was completely immersed in the play world.”; “...gave the caregiver clear, game-related instructions.”; “...understood what his role in the game was.”; “...had no problems with the controls of the game”; and “...was optimally challenged by the game” on a 7-point Likert scale.

##### Presence

According to our assumptions, playing a VR game creates presence, which can be explained as a redirection of the user’s attentional focus from the real to the virtual world. Hence, the experience of presence is an indicator of distraction from the medical situation. As there is no questionnaire designed for children to measure their sense of presence, we will use 5 items from the German Igroup Presence Questionnaire [41]. These 5 items describe how conscious the players were of the actual world and how real the virtual world seemed to them. The vocabulary used in these items is less abstract, which makes it easier for children to comprehend. The items are rated on a 7-point scale from 0 (strongly disagree) to 6 (strongly agree).

##### Simulator Sickness

Simulator sickness is a possible adverse side effect of VR exposure. To diagnose simulator sickness, we will use the Child Simulator Sickness Questionnaire adapted by Hoeft et al [42] and translated to German by us. The questionnaire comprises 7 items describing different symptoms summarized in dimensions “nausea,” “oculomotor,” and “disorientation.” Each symptom is rated on a 3-point scale ranging between 0 (“not at all”) and 3 (“very much”).

However, it should be noted that our target group will consist of children who are sick. Thus, it is likely that some participants, due to their illness, will experience symptoms that are also associated with simulator sickness. Therefore, we will ask the participants to rate their symptoms before (ie, before measurement) and after (ie, after measurement) the VR exposure.

#### Patient Experience

We will use several simple self-designed questions to assess specific aspects of the patient experience from the perspective of the children, the caregivers, and the interviewers, including a sense of control, perceived distraction from the medical situation, excitement, and surprise at the onset of pain.

#### Procedure Flow

The interviewers will be asked to evaluate the course of the medical procedure using the statement “Please evaluate the willingness of the patient and the accompanying person to cooperate regarding the examination procedure” (1=“very uncooperative”; 5=“very cooperative”). In addition, they will be asked to rate the overall procedure on a 5-point star scale. We will also track all premature terminations of the intervention and the reasons given by participants, if any.

### Data Collection and Analysis

All questionnaires will be completed electronically using the LimeSurvey (LimeSurvey GmbH) software to prevent data loss, incorrect data, or duplicate entries. Data will be stored anonymously on secured servers at the University Hospital Essen. The signed declarations of consent will be stored separately from the data collected. No identifying data will be stored in the dataset. The dataset will be stored separately from security-relevant patient data. Only the persons involved in the data analysis (SL and OB) will have access to the final dataset. Methods of descriptive and inferential statistics will be used to analyze quantitative data.

## Results

Data collection is ongoing at the time of publication of this study. As of November 2024, we enrolled 39 patients and caregivers, 28 of whom completed the study (Multimedia Appendix 2 presents the participant flow diagram).

## Discussion

### Overview

Although anxiety and pain at the hospital cannot always be avoided, there are situations in which the emotional experiences of patients and their caregivers can be improved in a controlled and safe manner using comparatively simple means. VR gaming has proven to be a suitable, effective measure for distracting pediatric patients in pain and anxiety-ridden situations and thus as a nonpharmacological analgesic. We take this approach one step further and involve the caregivers in the game. Therefore, our aim is to make the medical procedure as pain-, anxiety-, and stress-free as possible for patients and their companions to enable the most positive patient experience possible. With our intervention, we aim to increase the cooperativeness of patients and caregivers and thus optimize the course of the medical procedure in terms of minimizing health risks for the patients as well as the necessary time and personnel resources. In the study presented here, we use the VR game SDVR, a cooperative multiplayer game based on cooperation between patient and companion specially designed for this use case.

### Challenges

In our effort to enhance the experience for pediatric patients and their caregivers, we acknowledge the challenges associated with maintaining immersion and comfort during medical procedures, particularly when patients are visually and auditorily shielded from the real world; that is, some patients might feel uncomfortable when touched while visually and auditorily shielded from the real world [43]. In this context, it should be noted that there is currently no HMD that has been optimized for children’s heads. All existing devices on the market are designed for adults in terms of weight, dimensions, lens distance, and so forth. Consequently, patients may perceive the HMD as a strain and wish to discontinue wearing it during the procedure. In the study, we record such cases and the patients’ reasons.

Furthermore, unexpected real-world sensations (eg, phone calls, medical staff coming and going, and noises from medical equipment) that interfere with the virtual environment may “break” immersion and distraction [44]. Our approach addresses these concerns by integrating real-world stimuli into the VR gaming environment, thereby enhancing immersion and minimizing potential disruptions. By allowing the caregivers to trigger events in the game from the outside, real-world stimuli and game-related sensations can be aligned.

We might also encounter patients who are not eager to wear the HMD and engage in the game as they may perceive a sense of losing control over the situation. Previous adverse experiences with the same or similar medical procedures may have resulted in trauma and left patients so emotionally compromised that they perceive any additional treatment (ie, wearing the HMD) as an excessive burden. Reaching such patients with alternative interventions is generally very difficult.

As mentioned earlier, studies show that there is a relation between pain perception and age [16,17]. Against this background, the wide age range of our study is beneficial as it allows us to examine this relationship. In the results report, we will compare our findings with those of other studies to derive potential recommendations on the use of VR game–based pain distraction for pediatric patients of different age groups.

When designing our specific VR application SDVR, we already focused on seamless integration into medical treatment processes. Nevertheless, integrating VR into medical situations brings several challenges, and solutions and workflows must be found. These include the maintenance of the hardware (eg, system updates, battery charging, storage, and disinfection) but also the training of staff in the use of the technology. A VR solution can only provide real added value if it can be easily and safely integrated into the highly standardized processes of the hospital. It must be borne in mind that medical staff are primarily responsible for medical tasks, and experience has shown that, in many cases, they are already working to their capacity. In addition, previous experience and technological expertise cannot always be assumed. If a VR solution does not take these circumstances into account, there is a high risk that the technology will not be accepted and therefore not used. We tried to take these circumstances into account when developing SDVR and our intervention concept and want to use the planned study to check whether we have succeeded.

### Limitations

There are some restrictions regarding the study design and methodology. Answering self-report questionnaires about their emotional and perceptual experience can be difficult for younger children. Although we use instruments specially adapted and, where available, validated for children. The interviewers are trained to assist the children in answering the questionnaires without influencing their answering behavior. However, bias cannot be completely ruled out due to children’s psychoemotional and cognitive development including language skills, focus, and motivation. Sometimes the caregivers need to be kept from trying to influence the child when answering the questions. Given the nature of the matter, the study cannot be blinded. Therefore, it is not possible to fully objectify the interviewer’s assessment during the intraoperative observation.

The duration of exposure to the alleged anxiety- and pain-reducing stimuli (ie, SDVR and recording of ocean waves) cannot be standardized due to the variable length of the medical procedure. Therefore, it is not possible to determine whether the duration of the intervention influences the experience of patients and caregivers. In addition, the reality of daily practice in the hospital does not allow for a fully standardized, perioperative measurement of the relevant experience-related variables. Therefore, our study protocol defines realistic time frames before and after the procedure, representing a reasonable compromise between temporal proximity to the events and feelings under investigation, integration into hospital operational processes, and standardization with sufficient time for the participants to complete the questionnaires.

### Conclusions

This study represents a significant step forward in addressing the challenges of managing anxiety and pain in pediatric medical settings. By harnessing the immersive potential of VR gaming and actively involving caregivers in the process, we strive to create a supportive and stress-free environment for patients and their caregivers. Despite the complexity inherent in our presented study design, we are confident that our work contributes to advancing nonpharmacological interventions in pediatric health care.

## Appendix

Multimedia Appendix 1 Selection of items derived from the German version of the Player Experience Inventory used to assess player experience in children.

Multimedia Appendix 2 CONSORT (Consolidated Standards of Reporting Trails) participant flow diagram.

Multimedia Appendix 3 SPIRIT checklist.

## Abbreviations

HMD: head-mounted display
PXI: Player Experience Inventory
SDVR: Sweet Dive VR
VAS: visual analog scale
VR: virtual reality

## Data Availability

The datasets generated and analyzed during this study are available from the corresponding author on reasonable request.
