# Supplementary material for: Cooperative Virtual Reality Gaming for Anxiety and Pain Reduction in Pediatric Patients and Their Caregivers During Painful Medical Procedures: Protocol for a Randomized Controlled Trial
Source: JMIR Res Protoc. 2025 Mar 31;14:e63098. doi: 10.2196/63098 (PMC11997540; doi:10.2196/63098)
Supplement: Multimedia Appendix 1 [file resprot_v14i1e63098_app1.pdf]

Player Experience Inventory für Kinder (PXI-K)

Instruktion:

Bitte denke an das Spiel, das Du zuletzt gespielt hast. Denke dabei vor allem daran, wie Du Dich während des Spielens gefühlt hast.

Gib für jede Aussage an, wie gut sie auf Dein Empfinden zum Spiel passt. Du kannst von -3 bis +3 bewerten. Wenn Du -3 ankreuzt, trifft die Aussage überhaupt nicht zu. Wenn du +3 ankreuzt, trifft die Aussage voll und ganz zu.

Skala: 7-stufige Likert-Skala, von -3 (= Stimme überhaupt nicht zu) bis +3 (= Stimme voll und ganz zu)

1. Ich fand das Spiel war leicht zu bedienen.
2. Ich habe meine Aufgaben im Spiel verstanden.
3. Das Spiel war weder zu einfach noch zu schwierig.
4. Ich konnte meine Leistung im Spiel leicht einschätzen.
5. Mir gefiel das Aussehen des Spiels.
6. Das Spiel fühlte sich für mich wichtig an.
7. Ich war gespannt darauf, wie das Spiel weitergeht.
8. Ich hatte das Gefühl gut in dem Spiel zu sein.
9. Ich war voll auf das Spiel konzentriert.
10. Ich hatte das Gefühl, das Spiel so spielen zu können, wie ich es wollte.

HINWEIS

DIES IST EINE NICHT-VALIDIERTE VARIANTE DES ORIGINALEN Player Experience Inventorys (PXI) VON Abeele et al. (202) IN DER DEUTSCHEN FASSUNG VON Graf et al. (2022).

WEITERE INFORMATIONEN ZUM ORIGINALEN PXI SIND HIER ZU FINDEN:
https://playerexperienceinventory.org/

NOTE

THIS IS A NON-VALIDATED VARIANT OF THE ORIGINAL Player Experience Inventory (PXI) BY Abeele et al. (202) IN THE GERMAN VERSION BY Graf et al. (2022).

FURTHER INFORMATION ON THE ORIGINAL PXI CAN BE FOUND HERE:
https://playerexperienceinventory.org/

ORIGINAL RESEARCH

Abeele, V. V., Spiel, K., Nacke, L., Johnson, D., & Gerling, K. (2020). Development and validation of the player experience inventory: A scale to measure player experiences at the level of functional and psychosocial consequences. International Journal of Human-Computer Studies, 135, 102370. https://doi.org/10.1016/j.ijhcs.2019.102370

Vanden Abeele, V., Nacke, L. E., Mekler, E. D., & Johnson, D. (2016, October). Design and preliminary validation of the player experience inventory. In Proceedings of the 2016 Annual Symposium on Computer-Human Interaction in Play Companion Extended Abstracts (pp. 335-341). https://doi.org/10.1145/2968120.2987744

Linda Graf, Maximilian Altmeyer, Katharina Emmerich, Marc Herrlich, Andrey Krekhov, and Katta Spiel. 2022. Development and Validation of a German Version of the Player Experience Inventory (PXI). In Proceedings of Mensch und Computer 2022 (MuC '22). Association for Computing Machinery, New York, NY, USA, 265–275. https://doi.org/10.1145/3543758.3543763
