# Supplementary material for: Cooperative Virtual Reality Gaming for Anxiety and Pain Reduction in Pediatric Patients and Their Caregivers During Painful Medical Procedures: Protocol for a Randomized Controlled Trial
Source: JMIR Res Protoc. 2025 Mar 31;14:e63098. doi: 10.2196/63098 (PMC11997540; doi:10.2196/63098)
Supplement: Multimedia Appendix 2 [file resprot_v14i1e63098_app2.pdf]

Participant Flow Diagram

Cooperative Virtual Reality Gaming for Anxiety and Pain Reduction in Pediatric Patients and their Caregivers during Painful Medical Procedures: Research Protocol for a Randomized Control Trial

Stefan Liszio, Franziska Bäuerlein, Jens Hildebrand, Carolin van Nahl, Maic Masuch, Oliver Basu

Data collection as of: November 27, 2024*

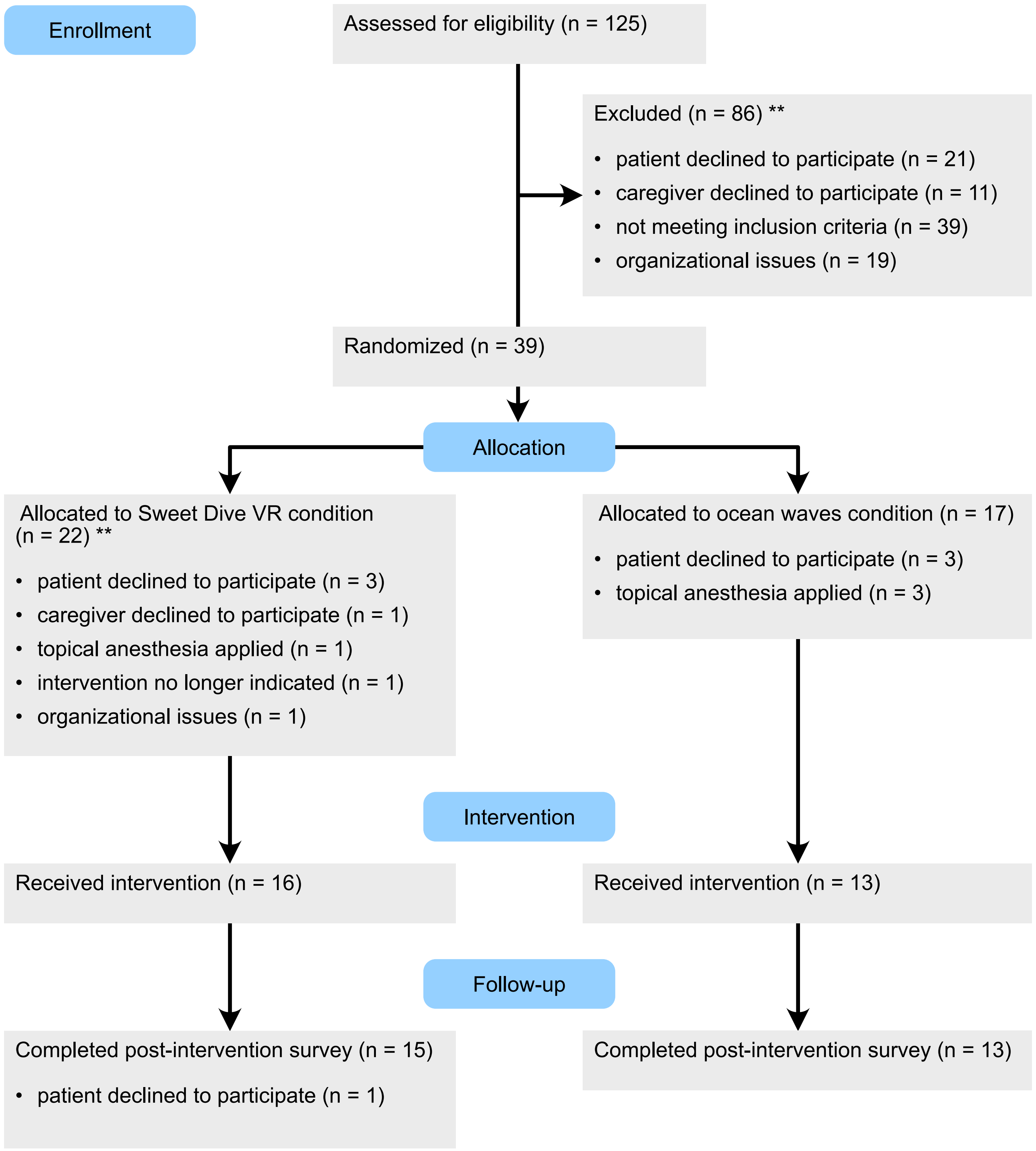

**Please note that the data collection process is ongoing at this time.*

*** Several reasons may apply.*
